# Association of the tissue infiltrated and peripheral blood immune cell subsets with response to radiotherapy for rectal cancer

**DOI:** 10.1186/s12920-022-01252-6

**Published:** 2022-05-09

**Authors:** Min Zhu, Xingjie Li, Xu Cheng, Xingxu Yi, Fang Ye, Xiaolai Li, Zongtao Hu, Liwei Zhang, Jinfu Nie, Xueling Li

**Affiliations:** 1 Anhui Province Key Laboratory of Medical Physics and Technology, Institute of Health and Medical Technology, Hefei Institutes of Physical Science, Chinese Academy of Sciences, 350 Shushanhu Road, Hefei, 230031 People’s Republic of China; 2 Institute of Physical Science and Information Technology, Anhui University, 111 Jiulong Road, Hefei, 230601 People’s Republic of China; 3 Hefei Cancer Hospital, Chinese Academy of Sciences, Hefei, 230031 People’s Republic of China; 4 Hefei Institute of Intelligent Machines, Hefei Institutes of Physical Science, Chinese Academy of Sciences, 350 Shushanhu Road, Hefei, 230031 People’s Republic of China

**Keywords:** Cancer immune microenvironment, gene expression profile deconvolution, Cancer infiltrated immune cell subset, Peripheral blood immune cell subset, Response to radiotherapy

## Abstract

**Background:**

Tumor microenvironment plays pivotal roles in carcinogenesis, cancer development and metastasis. Composition of cancer immune cell subsets can be inferred by deconvolution of gene expression profile accurately. Compositions of the cell types in cancer microenvironment including cancer infiltrating immune and stromal cells have been reported to be associated with the cancer outcomes markers for cancer prognosis. However, rare studies have been reported on their association with the response to preoperative radiotherapy for rectal cancer.

**Methods:**

In this paper, we deconvoluted the immune/stromal cell composition from the gene expression profiles. We compared the composition of immune/stromal cell types in the RT responsive versus nonresponsive for rectal cancer. We also compared the peripheral blood immune cell subset composition in the stable diseases versus progressive diseases of rectal cancer patients with fluorescence-activated cell sorting from our institution.

**Results:**

Compared with the non-responsive group, the responsive group showed higher proportions of CD4^+^ T cell (0.1378 ± 0.0368 vs. 0.1071 ± 0.0373, *p* = 0.0215), adipocytes, T cells CD4 memory resting, and lower proportions of CD8^+^ T cell (0.1798 ± 0.0217 vs. 0.2104 ± 0.0415, *p* = 0.0239), macrophages M2, and preadipocytes in their cancer tissue. The responsive patients showed a higher ratio of CD4^+^/CD8^+^ T cell proportions (mean 0.7869 vs. 0.5564, *p* = 0.0210). Consistently, the peripheral blood dataset showed higher proportion of CD4^+^ T cells and higher ratio of CD4^+^/CD8^+^ T cells, and lower proportion of CD8^+^ T cells for favorable prognosis. We validated these results with a pooled dataset of GSE3493 and GSE35452, and more peripheral blood data, respectively. Finally, we imported these eight cell features including eosinophils and macrophage M1 to Support Vector Machines and could predict the pre-radiotherapy responsive versus non-responsive with an accuracy of 76%, ROC AUC 0.77, 95% confidential interval of 0.632–0.857, better than the gene signatures.

**Conclusions:**

Our results showed that the proportions of tumor-infiltrating subsets and peripheral blood immune cell subsets can be important immune cell markers and treatment targets for outcomes of radiotherapy for rectal cancer.

**Supplementary Information:**

The online version contains supplementary material available at 10.1186/s12920-022-01252-6.

## Background

10% of estimated 19.3 million new cancer cases in 2021 are colorectal cancer, which is ranked 3rd, with a mortality rate of 9.4%, ranked 2nd, in estimated 10 million new cancer deaths [1]. Among the colorectal cancer, about 1/3 are rectal cancer. For the local advanced rectal cancer, the clinical guideline for treatment recommends preoperative chemoradiotherapy or radiotherapy (RT) as standard treatment [2]. Preoperative RT has advantages in down-staging tumor, increasing resectability, decreasing tumor viability, and possible sparing sphincter procedure. However, patients have different response to RT even classified as the same clinic TNM stages. Some patients’ local cancer may be under control without progression and metastasis, but this may not be the case for other patients [3].

The tumor microenvironment (TME) is the environment around the cancer cells within the cancer tissue, including the surrounding blood vessels, infiltrated immune cells, surrounding fibroblasts, etc. Cell types in cancer microenvironment include cancer infiltrating/resident immune cells and stromal cells [4]. Stromal cells mainly arise from pericytes, adipocytes, preadipocytes, endothelial cells, fibroblasts and myofibroblasts, etc. [5]. The cancer and its microenvironment are closely related and interact constantly, where interactions between cancer cells and other cell types elicit immune editing: immune elimination, immune equilibrium, and immune escape [6]. A hallmark of cancer is immune destruction or pro-tumor inflammation. A large amount of evidence demonstrates that cancer cells actively restrain or re-educate tumor-infiltrating or resident leukocytes (TILs) and stromal cells. Uncontrolled cancer cells induce anergy of T cells or apoptosis of activated T cells [7] and turn off the normal cytotoxic response of natural killer cells (NK cells) by secreting exosomes containing death ligands, such as FasL and TRAIL [8]. On the other hand, immune and stromal cells provide growth factors to support the survival of the cancer cells. Therefore, in vitro studies on cancer cells without considering the TME cannot necessarily reflect the in vivo response to cancer treatment.

Cancer immune microenvironment has recently been recognized to play an important role in the efficacy of RT [9, 10]. The cancer immune microenvironment is classified into infiltrated-excluded, infiltrated-inflamed, and infiltrated-TLS (tertiary lymphoid structure), which is usually associated with known or unknown cancer molecular subtypes and relates to therapy responses [11]. Tumor heterogeneity control the outcome of the radiotherapy treatment, where non cancer cells in the tumor environment can attribute to the resistance of the cancer de novo or recur with a worse prognosis following therapy by interacting with the cancer cells. Compositions of cell types in the cancer microenvironment, which represent the cellular level of inflammatory and immune circuit niche of cancer immunity, is related to the RT outcome [12]. Radiotherapy-mediated immunogenic cell death (ICD) elicited immune response may be limited by the presence of radioresistant suppressor cell types in the TME. Hypoxia plays a crucial role in radioresistance due to reduced oxygen-mediated DNA damage and hypoxia induced factor-1α (HIF-1α)-mediated cell survival. Attempts to prevent the recruitment of bone marrow derived cells (BMDCs) required for vasculogenesis are all being tested to reduce tumour hypoxia, improve radiotherapy responses and prevent tumour recurrence after therapy [13]. Antiagiogenisis drugs target endothelial cells and its interaction with tumor cells increase the radiosensitivity of tumors [14]. Increased tumor responses to neoadjuvant therapy were observed among rectal cancer patients taking angiotensin-converting enzyme inhibitors or angiotensin receptor blockers [15], possibly by vascular remodeling and modulating deregulated inflammation and macrophage activity [16]. In another study to the resistance to immune checkpoint blockade and to combinations of radiation plus ant-CTLA4, researchers found that IFGN drives high levels of PDL1 on both melanoma cells and CD45+ cells. The highest level was observed on tumor-associated macrophages among the immune cells, which augmented expression of interferon-stimulated genes and ligands for multiple T cell inhibitory receptors [17] in tumors. Tumor irradiation induces a wound healing response characterized by inflammation, cancer-associated fibroblast (CAF) modulation and ECM remodeling, which may facilitate tumor recurrence. Locally active neutrophils were key drivers of the tumor-supportive preconditioning of the lung microenvironment by enhanced regenerative Notch signaling [18]. Inflamatory fibroblasts mediate resistance to neoadjuvant therapy in rectal cancer [19]. A recent study [20] reports that a large portion of cancer resident T cells survives the clinically relevant radiation dose. Compared with the circulating T cells, cancer reprogrammed T cells are more resistant to radiation. The cancer-associated T cells can lead to a response to RT without need of recruiting new or additional infiltrating T cells. These T cells experienced reprogramming in the cancer microenvironment and resembled tissue-resident memory T cells in transcription profiles. The up-regulation of the TGF beta regulator conferred this reprogramming and resistance to radiation [20].

Although immune cells present in blood might not reflect directly the components of the TME, its presence might indirectly reflect the regulators or modulators present in TME [21]. Indeed, some studies have correlated some inflammatory biomarkers, such as T cells, DC or NK cells, in blood and tumor tissue samples and found a good correlation between both sites [22, 23]. Resident and circulating memory T cells persist for years in melanoma patients with durable responses to immunotherapy. Paired T-cell receptor sequencing identified dispersed clonotypes throughout tumor, skin and blood highly expressed IFNG/TNF signature and have a strong prognostic value for patients with melanoma. Clonotypes from tumors were found in patient skin and blood up to 9 years later [24]. In another study, circulating immune cell phenotype dynamics, including increased cytotoxic differentiation and strong activation of interferon signaling in peripheral T cells in responder patients reflected the strength of the tumor-immune cell interactions in patients during immunotherapy [25]. Increased expression of CD161+ on CD4+ T cells, which may represent a specific subpopulation of T_H_17, was seen in papillomavirus-related cervical carcinoma patients with progressive disease, but not in patients with partial response or stable disease [26]. Peripheral white blood cell subsets in metastatic colorectal cancer patients treated with cetuximab have a potential clinical relevance to the response to therapy [27].

With the progress of high throughput technology, gene expression profiles of cancer tissues accumulated rapidly. Cell type deconvolution overcomes the limited small number of cell type-specific markers of the fluorescence-activated cell sorting (FACS) and immunohistochemistry (IHC) on investigating the cell type composition of cancer tissue. Cell type composition of the cancer tissues can be accurately inferred from the deconvolution of gene expression profiles [28–30]. Prediction of the responsiveness of rectal cancer to radiotherapy will help patients to gain greatest benefits from the RT. Sensitivity of cancer cells to RT [31, 32] and efficient doses[33] have been reported based on genomic or genetic molecular radiosensitivity markers [32, 34–36] from cancer cell lines. These approaches may be more accurate by considering the cell type compositions of the tumor microenvironment in predicting cancer type-specific outcomes of RT.

Cytolytic activity [37] has been reported to be important for cytolytic T cells’ function in anti-cancer immunity and clinical outcomes in colorectal cancer [38]. However, the respective contribution of the statistically significant differential cell types to the overall cytolytic activity is unknown. Less is known whether it is associated with outcomes of rectal cancer radiotherapy, and if yes, favorable or unfavorable. By evaluating the correlation of significant tissue-resident immune/stromal cell fractions and cytolytic activity markers, and comparing the expression levels of representative cytolytic activity markers, cytokines and chemokines important in cancer immunity in RT responsive and non-responsive patients, we explored these questions.

This study uses deconvolution methods to infer the cell type composition and the immune/cytolytic activity from the gene expression profile of patients with rectal cancer before preoperative radiotherapy. We use statistical tests and machine learning to study the relationship between the cell type composition of rectal cancer and the outcome of RT. Furthermore, we explore the relationship between the composition of peripheral blood white blood cell subsets and the prognosis of rectal cancer. We validated our results on combined datasets. Our results will provide potential markers of infiltrating/resident leukocytes (TIL) and stromal cells in the prognosis of rectal cancer with RT. Our research may provide inspiration for the development of new sensitization or immunotherapy combinations for rectal cancer radiotherapy.

## Methods

### Gene expression profiles

We downloaded the microarray expression profiles (GSE3493 https://www.ncbi.nlm.nih.gov/geo/query/acc.cgi?acc=GSE3493) of rectal cancer from Gene Expression Omnibus (GEO). The dataset includes 46 samples consisting of 35 nonresponsive and 11 responsive patients. As reported in [34], response to RT was determined by histopathologic examination of surgically resected specimens based on a semiquantitative classification system as described in [39]. Tumors were classified as “responder” when assigned to the regression grade 2 or 3, and “nonresponder” when grade 0 or 1. Grade 0 and Grade 1 were assigned when there were no (Grade 0) or less than two-thirds (Grade 1) tumor cell necrosis or degeneration observed in response to treatment, respectively. Grade 2 and 3 were assigned when prominent tumor cell necrosis, degeneration, lytic change, and/or disappearance present in more than two-thirds (Grade 2), and throughout (Grade 3) the entire lesion without viable tumor cells observed. Assessment was performed on as many pathological specimens as possible, including those prepared from the section of the whole tumor at its largest diameter [40]. The downloaded data were in normalized data file format for direct analysis unless otherwise stated. When multiple probesets correspond to one gene, we averaged expression levels of the probesets for each unique gene.

### Cell type deconvolution and statistical analysis

To deconvolute the rectal cancer tissue expression profiles (GSE3493) into cell type composition, TIMER (Tumor Immune Estimation Resource) [29], CIBERSORT [30] and xCell [28] were run with default parameters and signature matrices. To keep the statistical power, we only kept the stromal cells inferred by the xCell while kept all the TILs inferred from TIMER and CIBERSORT and resulted in 40 cell types. The proportion of each cell type between RT responder and non-responders was tested by two-sample *t*-test function with the equal or unequal variance, or a Wilcoxon rank sum test (Mann–Whitney U-test) if appropriate (Matlab). The variances of clinical parameters were tested by the Chi-square variance test between RT responders and non-responders if necessary.

### Stratification of T cells into cytotoxic, exhausted and inflammatory categories and differential analysis

We further stratified the T cells into cytotoxic, exhausted, and inflammatory subtypes. Activated CD8+ T cells and nature killer T cells represent the T cells cytotoxic. Th17, Th22 cells and CD8 T effector memory cells represented the inflammatory subtypes. We collected cell markers from [41] for cytotoxic and inflammatory T cell subtypes and [42] for exhausted CD8+ T cells, respectively, and applied single sample Gene Set Enrichment Analysis [43]. We then compared the enrichment scores of the gene markers of each cell types and performed differential analysis between responders and non-responders on GSE3493.

### Validation of the cell type statistical analysis results

To validate the cell type statistical analysis results from GSE3493, we downloaded another dataset of rectal cancer gene expression profiles before chemoradiotherapy (GSE35452, https://www.ncbi.nlm.nih.gov/geo/query/acc.cgi?acc=GSE35452). We performed the cell type deconvolution as procedures mentioned above and pooled the deconvoluted cell type result with that from radiotherapy dataset GSE3493, resulting in 92 samples with 35 responders and 57 non-responders. Differentially analysis between the two groups were performed as above.

### Cytolytic activity calculation

Gene expression levels of cytolytic activity including GZMA, GZMB, and PRF1, cytokine IFNG, and chemokine CXCL10 were compared between responsive versus non-responsive rectal cancer patients based on *t*-test with equal and unequal variance or a Wilcoxon rank sum test (Mann–Whitney U-test) if appropriate. A *p* value < 0.05 was considered significant. Similarly, cytolytic activity (CYT) score [44] was calculated as the geometric mean of *GZMA* and *PRF1* and compared between responsive and non-responsive rectal cancer patients.

### Correlation analysis between the significant cell type proportions and the cytolytic activity

Association studies of the significant infiltrating immune/stromal cell fractions with the cytolytic/immune activity molecular markers were performed based on the gene expression correlation pair-wise analysis across all patients. The analyzed significant cell types consist of CD4^+^ T cells, CD8^+^ T cells, preadipocyte, adipocyte, T cells CD4 memory resting, and macrophages M2. The cytolytic/immune activity molecular markers include GZMA, GZMB, PRF1, INFG, and CXCL10. For this analysis, we used the Matlab function *corr*(*C*, *M*, “type”, “kendal”) of the kendal rank level correlation. *C* represents the matrix of the immune/stromal cell compositions with columns of cell types and rows of patient samples. *M* represents the matrix of gene expression profiles of cytolytic/immune activity molecular markers as columns and patient samples as rows. Similarly, the pair-wise correlation analysis of the significant infiltrating/resident cell fractions between themselves were done by Matlab function *corr(C, C,* “type”, “kendal”*)* of the Kendal tau coefficient, where the *C* represents the matrix of the cancer tissue-resident immune/stromal cell compositions with columns of cell types and rows of patient samples. The function *corr* output a r value and a *p* value. A *p* value < 0.05 was considered significant.

### Prediction of the RT outcomes

**Fig. 1:**
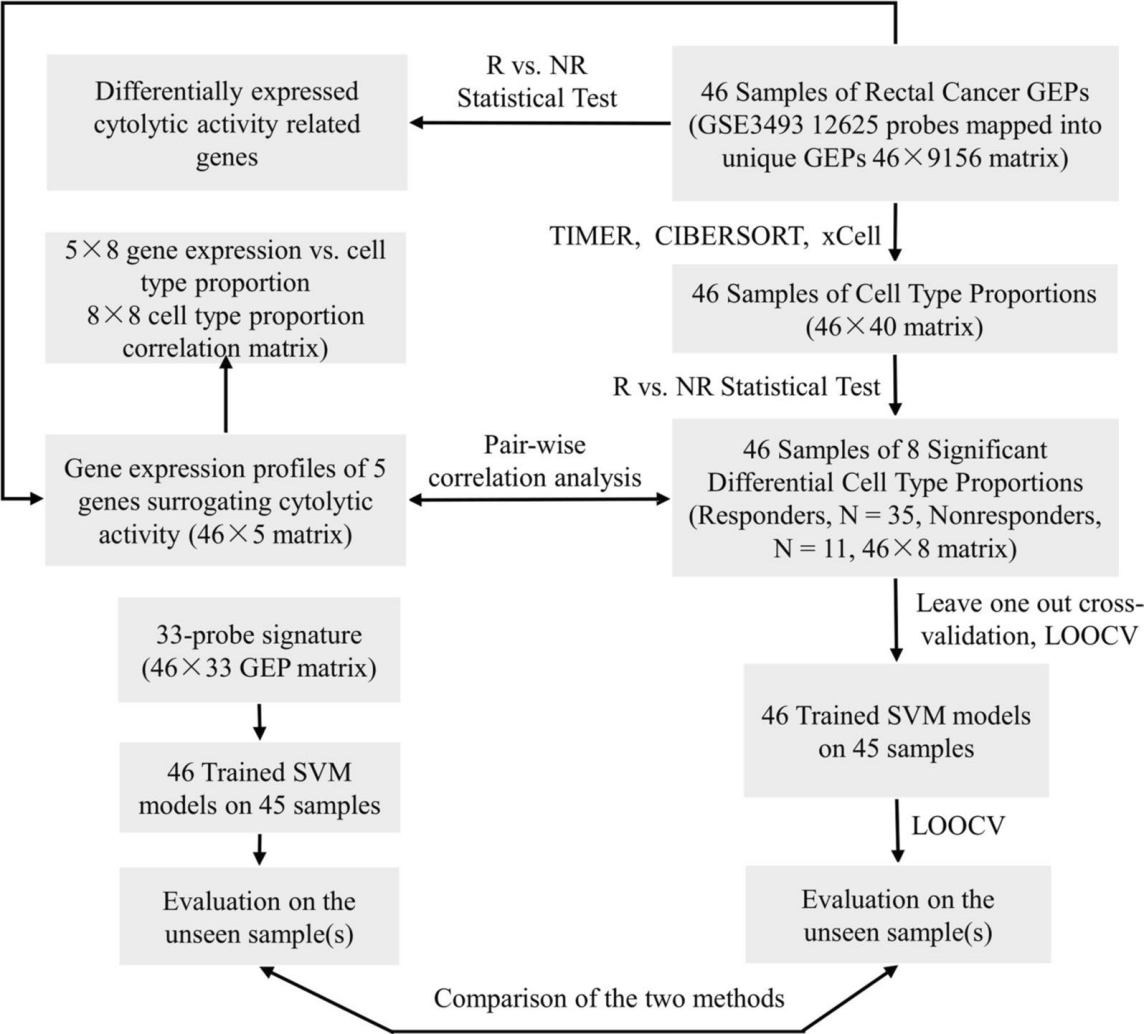
The workflow of this study

The 11 RT responsive patients were defined as positive samples and the 35 non-responsive patients were defined as negative samples as mentioned above in this section [34]. The significant cell proportion values were put together as the input vector for the support vector machine classifier. The *fitcsvm* and *predict* function in Matlab with default parameters were adopted in the training, leave one out cross-validation and independent assessments. Each significant cell type proportion corresponds to one element of each input vector, and all the proportions compose all the elements of each vector for each patient. Support vector machines (SVM) were trained on the original training dataset with one hold-out sample and evaluated with leave one out cross-validation (see Fig. 1). The influence of the imbalance of the positive and negative samples was mitigated by quadrupling the 11 responsive samples.

The performance of the above eight-cell-type-proportion predictors for the RT outcomes of the rectal cancers was compared with previously reported 33-gene radiosensitivity signature [34]. SVM was used as the common classifiers to ascertain an objective comparison, which is suitable for the small sample dataset in this study. The cell type proportions and gene expression profiles of the 33 genes, respectively, composed the input vectors. Leave one sample out cross-validation (LOOCV) was used to estimate the performance of the SVM models. The accuracy, ROC, true positive rate (*TPR*), false positive rate (*FPR*), specificity, sensitivity, and F-score were respectively calculated and compared. Specifically, the true positive rate equals the number of true positives (*TP*) over the sum of the true positives (*TP*) and false negatives (*FN*), i.e., *TPR* = *TP*/(*TP* + *FN*). The false positive rate equals the number of false positives (*FP*) over the sum of the number of false positives (*FP*) and true negatives (*TN*), i.e., *FPR* = *FP*/(*FP* + *TN*). The specificity equals *1-FPR*, while the sensitivity (or precision, *PE*) equals *TP*/(*TP* + *FP*), and the F-score is 2 × *PE* × *TPR*/(*PE* + *TPR*).

A recent report [45] demonstrated that both nested CV and parameter tuning partially nested cross-validation produced robust and unbiased performance estimates regardless of the small sample size for SVM. The SVM nested CV and partial nested CV are defined, respectively, as feature selection (*t*-test in this study) on pooled training and test data and on training data only. The performance was estimated by LOOCV as above.

### Fluorescence-activated cell sorting data of peripheral blood cells

Data of the proportions of the peripheral blood immune cell subsets from fluorescence-activated cell sorting (FACS) of rectal cancer patients hospitalized from 2018 through 2019 were downloaded from our institution system (The Center of Medical Pathology, Hefei Cancer Hospital, Chinese Academy of Sciences). Briefly, fasting venous blood was drawn before inpatient treatment into anticoagulant EDTA-K2 tubes. 20 µL of CD3/CD8/CD45/CD4 mono-antibody kit solution were added to the bottom of the FACS tube, and then 50 µL of fasting venous blood was added to the bottom of the FACS tube avoiding touching the wall of the FACS tube. The tube cap was screwed, vortexed for 3 s, and incubated for 15–25 min at room temperature. 1 mL buffered ammonium chloride (ACK) solution was added to lyse the red blood cells. Tube cap were then screwed, vortexed for 10 s, and incubated for 10 min at room temperature. Next, 4 mL of buffered PBS solution were added and centrifuged for 5 min at 500*g* to wash the leukocytes. The supernatant was discard quickly to avoid cell loss with about 200 µL cell solution left and the solution were mixed with vortex for 3 s. The above wash steps were repeated once and finally 200 µL PBS buffer were added to suspend the cells, which was ready for flow cytometer (Beckman Counter Biotechnology, Suzhou Co., Ltd). Beijing Datong Biotech Company LTD provided all the reagents. Treatment records, prognostic and pathological parameters were manually collected retrospectively from the Hospital Information System (HIS). Patients were grouped into RT and chemotherapy. According to response to therapies, patients were further grouped into progressive disease and stable disease, respectively, based on the Response Evaluation Criteria in Solid Tumors (RECIST v1) criteria.

To validate the results from the above blood dataset, we merged the peripheral blood data of patients hospitalized in 2020 and 2021 with the above blood dataset of 2018 through 2019. We then performed the same analysis on the data of all rectal cancer patients, including pre-chemotherapy, pre-radiation and pre-chemoradiation rectal patients.

## Results

### Differential tumor immune/stromal cell proportions in RT responders versus non-responders

**Fig. 2:**
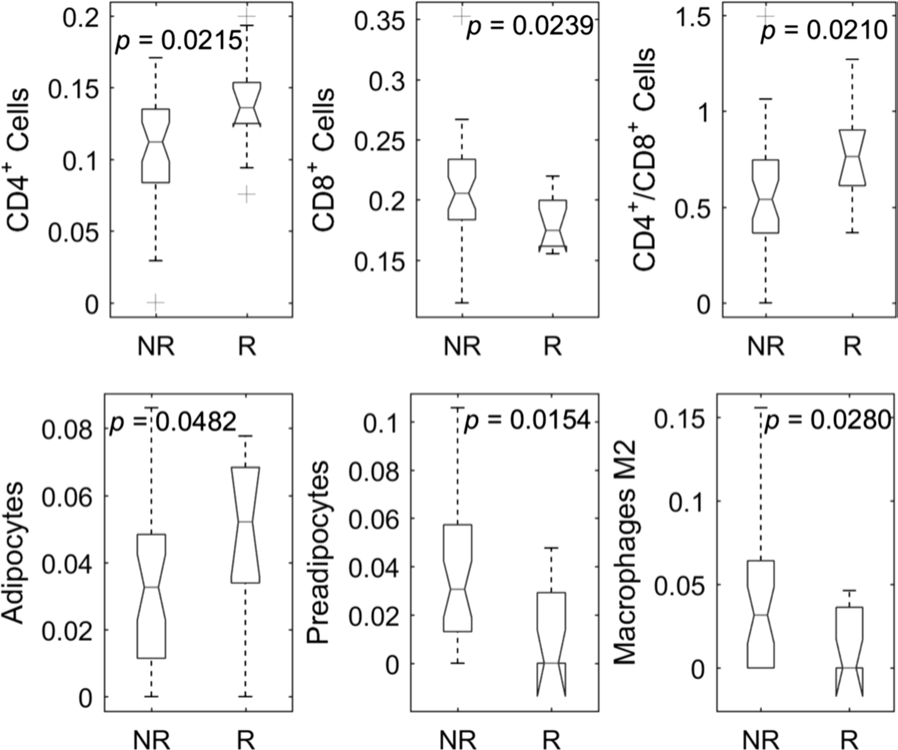
Representative cell subtype proportions with *p* < 0.05 between responders (R) versus. non-responders (NR) to RT for rectal cancer patients by using student *t*-test with equal or unequal variance if appropriate

Eight of forty tested cell proportions were significantly different in responders (R) versus non-responders (NR) of RT for rectal cancer patients. Two (The proportion values of macrophage M1 and eosinophils) were zeros in most samples (see Additional file 1). Among the remaining six statistically significant cell types (see Fig. 2 and Table 1), three cell types had higher proportions in R versus NR. These cell types were CD4^+^ T cells (mean proportion R vs. NR: 0.1378 vs. 0.1071, *p* < 0.05, see Table 1), adipocytes (mean proportion R vs. NR: 0.0479 vs. 0.0314, *p* < 0.05) and T cells CD4 memory resting (mean proportion R vs. NR: 0.0864 vs. 0.0293, *p* < 0.05). Three cell types had lower proportions in R versus NR. These cell types were CD8^+^ T cell (mean proportion R vs. NR: 0.1798 vs. 0.2104, *p* < 0.05), preadipocyte (mean proportion R vs. NR: 0.0129 vs. 0.0363, *p* < 0.05), and macrophage M2 (mean proportion R vs. NR: 0.0144 vs. 0.0346, *p* < 0.05, see Table 1). The ratio of CD4^+^/CD8^+^ T cell proportions was significantly higher in R versus NR (0.7869 vs. 0.5564, *p* = 0.021).

**Table 1:** Significant differential proportions of tumor immune/stromal cell types between the non-responsive and responsive rectal cancer tissues

Cell types	Methods	(R) Mean ± SD	(NR) Mean ± SD	*p* value	Variance type
CD4^+^ cells	TIMER	0.1378 ± 0.0368	0.1071 ± 0.0373	0.0215	Equal
CD8^+^ cells	TIMER	0.1798 ± 0.0217	0.2104 ± 0.0415	0.0239	Equal
				0.0031	Unequal
CD4^+^/CD8^+^ ratio	TIMER	0.7869 ± 0.2696	0.5564 ± 0.2813	0.0210	Equal
Preadipocytes	xCell	0.0129 ± 0.0195	0.0363 ± 0.0286	0.0154	Equal
Adipocytes	xCell	0.0479 ± 0.0249	0.0314 ± 0.0230	0.0210	Equal
T cells CD4 memory resting	CIBERSORT	0.0864 ± 0.1149	0.0293 ± 0.0583	0.0330	Equal
Macrophages M2	CIBERSORT	0.0144 ± 0.0199	0.0346 ± 0.0380	0.0280	Unequal

SD: standard deviation; (R): propotion of respornsive; (NR): proportion of non-respondive

### Stratification of T cell subtypes

We further stratified the T cells into cytotoxic, exhausted, and inflammatory subtypes. The representative T cell cytotoxic include activated CD8^+^ T cells, CD8 T effector memory cells and nature killer T cells. The inflammatory subtypes include Th17 and Th22 cells. We collected cell markers from [41] for cytotoxic and inflammatory T cell subtypes and [42] for exhausted CD8+ T cells, respectively, and applied single sample Gene Set Enrichment Analysis [43]. As detailed in Methods section, we then performed differential analysis of responders versus nonresponders for the functional categories on GSE3493. We found no statistical significant cell subtypes except lower activated CD8^+^ T cells in RT responsive groups(*p* = 0.038), which was consistent with the lower CD8^+^ T cells from TIMER.

### Validation of the cell type statistical analysis results

Microarray data are known for having larger data noise thus requiring a larger sample sizes to perform prognostic analysis. To validate the results of the cell type statistical analysis from GSE3493, we downloaded another dataset, GSE35452, of rectal cancer pre-chemoradiotherapy. We performed the cell type deconvolution separately and pooled the deconvoluted cell type result with that from radiotherapy dataset GSE3493, resulting in a relatively bigger dataset of 92 samples with 35 responders and 57 non-responders. Differentially analysis between the two groups demonstrate five of the six statistically significant cell types overlapping. Specifically, CD8+ T cell, preadipocyte, adipocyte, fibroblast, macrophage M2 in Table 1 were also found statistically significant from the pooled data (see Additional file 2). Although CD4+ T cell is not significant statistically, its main subset Th1 is. The difference may result from the treatment difference between the two datasets. The results demonstrated these cell types may be involved in regulating the response of rectal cancer to radiotherapy.

### Correlation analysis of significant tissue-resident immune/stromal cell fractions and the tumor/cancer local cytolytic/immune activity molecular markers

We analyzed previously well-characterized cytolytic/immune activity related genes corresponding to granzyme A (GZMA), granzyme B (GZMB), perforin (PRF1) and interferon-gamma (IFNG) and CXCL10. Chemokine 10 (CXCL10) attracts leukocytes to infiltrate the cancer tissue. Gene expression levels of GZMA, GZMB, PRF1, IFNG and chemokine CXCL10 were compared between responsive versus non-responsive rectal cancer patients. Unexpectedly, GZMA was significantly lower in responsive group (R vs. NR, 43.3 ± 21.1 vs. 90.9 ± 89.6, *p* = 0.006, t-test with unequal variance). We further calculated the cytolytic activity (CYT) as represented by the geometric mean of the expression of GZMA and PRF1 gene. Similarly, the CYT was significantly higher in nonresponders than in responders (143.4 ± 77.5 vs. 99.4 ± 42.4, *p* = 0.022). Correlation analysis was performed between each proportion of significant differentially distributed cell types and the gene expression levels of each surrogate gene across patients. As stated in Methods section, *corr* function output a *r* value, representing the extent of the correlation, where a higher *r* absolute value means a greater extent of the correlation and the sign of *r* represents the positive or negative correlation, and a *p*-value indicating the significance of the correlation. A p-value < 0.05 was considered significant and marked bold in Table 2. Results showed that CD4^+^ T was most significantly and positively associated with interferon-gamma gene (IFNG) expression, consistent with their favorable role in predicting RT outcome (see Table 2). As unfavorable predicting factors for RT outcome, proportions of preadipocyte were most negatively associated with perforin gene expression and proportions of macrophage M2 negatively correlate with PRF1 and IFNG but not reach the significant level. CD8^+^ T cellsdid not show a significant correlation with the expression of any of these five genes, only with marginal significant with PRF1 and INFG. Adipocyte and T cells CD4 memory resting were negatively and significantly associated with IFNG and PRF1 expression, respectively.

**Table 2:** Correlation analysis between significant cell type proportions and cytolytic activity molecular signatures

Correlation	PRF1		IFNG	
	*r*	*p*	*r*	*p*
CD4^+^ T cells	− 0.018	0.865	**0.341**	**0.00070**
CD8^+^ T cells	0.192	0.061	− 0.194	0.058
Preadipocyte	**−** **0.236**	**0.023**	− 0.097	0.352
Adipocyte	− 0.152	0.147	**−** **0.224**	**0.033**
T cells CD4 memory resting	**−** **0.221**	**0.049**	− 0.019	0.873
M2	− 0.211	0.087	− 0.227	*0.065*

*r,* correlation coefficient; *p*, significance of the correlation

### Correlation analysis of significant tissue-resident immune/stromal cell proportions between themselves

We performed the pair-wise correlation analysis on each significant tissue-resident immune/stromal cell fraction across patients. The results were demonstrated in Table 3. A *p*-value < 0.05 was considered significant and marked bold in Table 3. Results showed that CD4^+^ T cells had the most significant and negative correlation with CD8^+^ T cells, consistent with their genesis process and their roles in predicting RT outcome. However, the proportion of adipocytes as a favorable factor had a negative correlation with that of CD4^+^ T cells. This negative relationship may be irrelevant to their function in mediating the outcome of RT. Preadipocyte was relatively most independent as suggested by no significant association with any of the other significantly and differently distributed cell types. Contrary to their roles in predicting RT outcome, T cell CD4 memory resting was significantly and positively associated with macrophage type 2 (M2), which may be irrelevant to their functions in the RT efficacy.

**Table 3:** Correlation analysis between the proportions of significant cell types

Correlation	CD4^+^ T cells	CD8^+^ T cells	Pre-adipocyte	adipocyte	T cell CD4 memory resting
CD8^+^ T cells					
*r*	**−** **0.544**				
*p*	**2.00E−8**				
Adipocyte					
*r*	**−** **0.222**	0.033	0.030		
*p*	**0.034**	0.760	0.781		
M2					
*r*	0.092	− 0.087	0.176	− 0.089	**0.346**
*p*	0.463	0.491	0.160	0.488	**0.010**

*r,* correlation coefficient; *p,*significance of the correlation

### Immune cell type based RT response prediction

The two cell types of our previously mentioned eight significant cell types, i.e., macrophage M1 and eosinophils deconvoluted by CIBERSORT are zero inflated in our dataset, which is not suitable for t-tests as suggested. However, a truncated rank sum test is suitable to these inflated non-negative samples [46]. CIBERSORT has been reported to have a detection limit of 1% for samples with tumor content greater than 50% and 0.1% for samples with tumor content less than 50% [30]. We consider the first scenario where the tumor content is greater than 50% with 1% detection limit of a cell type proportion. Considering the non-zero-samples are all greater than 1% (see Additional file 1) with three exceptions of 0.76%, 0.4% and 0.18%, and those with proportions greater than 1% are not equally distributed in RT responders versus non responders. Specifically, all the non-zero-samples great than 1% for macrophage M1 are from 35 RT non-responders with an averaged value of 2.55%, while non-zero samples for eosinophils are all greater than 1% and from 11 RT responders, although only marginally significant as tested by using a truncated rank sum test duet to our sample size. Although the cell types macrophage M1 and eosinophils only identified in a very few samples, they are differentially distributed. Our previous study suggested that these features provided information for SVM prediction, or at least was not harmful for the prediction (data not shown here), with a discretization like effect, which is usually helpful in removing noise for machine learning methods. We still included the proportions of these two cell types as the input into the SVM prediction.

Prediction of the RT outcome was performed and evaluated as shown in the flowchart of Fig. 1. Support vector machine (SVM) classifiers were used to classify patients into responsive and non-responsive groups based on the proportions of cell types. Thus, eight tumor-infiltrating immune cell proportions inferred from gene expression deconvolution were used as the input (Additional file 3, Table S1). Leave one out cross-validation (LOOCV) was used to estimate the SVM performance. We achieved an accuracy of 72.15% and an AUC of 0.7610. By centralizing the training variables with a mean of zero and unit variance, we further increased the AUC to 0.771, decreased the false positive rate, and increased in Specificity, Precision, and F-score (see Table 4). We then compared the performance of the eight significant cell type proportions as markers with previously reported 33-probe signature on predicting the response to the RT for rectal cancer based on the same SVM model. Results showed a comparable or better predictive performance of the eight-cell-type composition markers (Additional file 3: Tables S1–S2). Moreover, cross-validation (CV) of both the SVM partial nested and completely nested CV [45] resulted in similar performance (see Additional file 3: Table S3), suggesting no over-fitting for evaluating the performance of predictors and the classifier in this study.

**Table 4:** Predictive performance of the cell type proportions based on support vector machine classifiers with balanced data of RT

Performance	8 compositions	Z-score of 8 compositions
Accuracy	0.722	0.759
AUC	0.761	0.771
TPR	0.727	0.727
FPR	0.286	0.200
Specificity	0.714	0.800
Precision	0.762	0.821
F-score	0.744	0.771

### Association of peripheral blood circulation leukocyte subpopulation and the outcome of RT

Patient**s** were grouped according to response to therapies (see “Method” section). We compared the average levels of peripheral blood circulation leukocyte subsets in patients with progressive disease (PD) versus stable disease (SD) by using the student’s *t*-test of equal variance for RT. The compared groups included six samples of stable disease and one incomplete response versus seven of progressive disease in RT. The compared peripheral blood circulation leukocyte subpopulation included CD3-CD19+ (B cells), CD3+ CD4+ (CD4^+^ T cells), CD3+ CD8+ (CD8^+^ T cells), CD3-CD16+ CD56+ (nature killer cells), CD3^+^ cells (CD4^+^ T cells plus CD8^+^ T cells), and ratio of CD4^+^/CD8^+^ T cells. Our results showed that CD4^+^ T cells and CD4^+^/CD8^+^ T cell ratio were significantly higher on average for better prognostic in RT (Table 5). We noticed a significant over representation in age older than 60 in progressive disease (Additional file 3: Table S4). We further examined if there was a significant difference between the patients older than 60 and younger than 60. Results showed no significant difference in CD4^+^ T cells and CD4^+^/CD8^+^ T cell ratio and other tested cell types (Additional file 3: Table S5), indicating the significant difference was not caused by the difference of age. This was consistent with the favorable prognostics of a higher level of CD4^+^ T cells and CD4^+^/CD8^+^ T cell ratio infiltrated into the rectal cancer tissue. Our result demonstrated that the tissue infiltrating immune cell types and the blood circulation leukocyte subpopulation had consistent prognostic values, suggesting the possible association between tissue infiltrating immune cell composition and blood circulation immune cell subset composition for radiotherapy response.

**Table 5:** Comparison of the proportions of peripheral immune cell subsets between the evaluated progressive and stable rectal cancer patients of RT

RT	*p* value	Progresive (mean ± sd)	Stable (mean ± sd)
CD3−CD19+	0.502	6.53 ± 4.22	7.36 ± 3.26
CD3+ CD4+	0.00569	21.2 ± 13.7	44.4 ± 12.3
CD3+ CD8+	0.941	27.5 ± 8.5	25.6 ± 6.49
CD3−CD16+ CD56+	0.0634	28 ± 11.4	17.3 ± 9.81
CD3+	0.0284	60.5 ± 12	73.2 ± 7.83
CD4+/CD8+	0.0254	0.83 ± 0.566	1.88 ± 0.803

For comparison, we also compared nine patients with progression disease versus eight patients with stable diseases in chemotherapy. The statistical characteristics of the rectal chemotherapy patients showed a balanced age, sex and cancer stages (see Additional file 3: Table S6). Lower levels of CD8^+^ T cells and CD3^+^ T cells, and a higher level of NK cells (CD3^−^CD16^+^CD56^+^) were better prognostic markers in chemotherapy (Additional file 3: Table S7). The results indicate that composition of the peripheral immune cell subset have different association with prognostics of different treatment modalities.

We validated the prognosis result with a total number of 255 blood samples before or during the beginning of regular treatment in our hospital for rectal cancer to mitigate the effect of the small number of samples. Characteristic of the patients are shown in Additional file 3: Table S8 with balanced age and sex distribution. CD4 over CD8 ratio is still a favorable factor for the rectal cancer prognosis (Additional file 3: Table S9). The results indicate that CD4/CD8 ratio may be a marker for rectal cancer prognosis.

## Discussion

We studied the association of the immune/stromal cell type compositions in the tumor microenvironment with the outcome of RT cancer treatment. Combing the results of the cell type proportions from three well-known cell type deconvolution methods, TIMER, CIBERSORT, and xCell may uncover significant cell types that each method cannot. Six cell types were found significant by comparing the 40 cell type proportions in responsive (R) versus non-responsive (NR) rectal cancer tissues, i.e., CD4^+^ T cells, adipocyte, and CD4 memory cells resting as favorable prognosis factors, while CD8^+^ T cells, preadipocyte, and macrophage M2 as unfavorable prognosis factors. Our results are reminiscent of a recent study which shows the more radioresistant infiltrating resident immune cells than their circulating counterparts may elicit the efficacy of the RT treatment for the cancers [20].

### Higher CD4^+^/CD8^+^ ratios are favorable in response to RT for rectal cancer

This study showed favorable CD4^+^/CD8^+^ ratios and unfavorable CD8^+^ T cell component in predicting the response of preoperative RT for rectal cancer. Consistently, Diederichson, et al. (2003) showed a higher ratio of tumor infiltrating CD4^+^/CD8^+^ T cells predicts a higher 5-year survival rate independent of Dukes stage and age from 41 cases [47]. Our study show reverted ratios of CD4^+^/CD8^+^ T cells in rectal cancer tissues and progressive disease (R vs. NR: 0.787 vs. 0.556, *p* < 0.05; progressive vs. stable: 0.83 vs. 1.83, *p* < 0.05) than normal value of around 2.0, which were also observed in cervical cancer [48] and breast cancer [49]. Conflicting results also showed that high density of CD4^+^ and CD8^+^ T cell in tumor were independent favorable prognostic factors for chemoradiotherapy [50] on 48 cases of rectal cancer. We propose that the discrepancy is due to different treatments. The unfavorable higher CD8^+^ T cells for radiotherapy for rectal cancer may be due to the cancer-educated properties of these special infiltrated CD8^+^ T cells, which is very different from the normal CD8^+^ T cells in function.

The favorable CD4^+^ T cells and unfavorable CD8^+^ T cells in predicting preoperative RT response of rectal cancer may relate to the much higher frequency of tumor-specific MHC class II epitopes versus MHC class I epitopes, and the relative paucity of the dendritic cells more required for the priming of CD8^+^ T cells. Specifically, CD8^+^ T cells are MHC class I dependent, which is frequently downregulated in tumor immune evasion since it is essential for CTL-mediated tumor elimination [51]. CD4^+^ T cells are MHC class II dependent. MHC Class II expresses at various levels in cancers and can be inducible. A recent study showed that spontaneous and immunotherapy-induced anti-tumor responses require the activity of tumor-antigen-specific CD4^+^ T cells, even in tumors that do not express major histocompatibility complex (MHC) class II molecules, which may be relevant with our finding that the higher level of CD4^+^ T cells was beneficial in RT responders of rectal cancer prognostics. Actually, researchers reported the cytolytic CD4^+^ T cells mediated immunity against cancer [52].

### Adipocytes and T cell CD4 memory resting are associated with favorable prognosis, but preadipocytes and tumor-associated macrophagemacrophage M2 are associated with unfavorable prognosis

We showed that adipocytes and preadipocytes as stromal cell types were favorable and unfavorable signs, respectively, for RT outcomes. One study reported that the direct interaction between adipocytes and epithelial cancer cells promoted phenotypic changes of cancer-associated adipocytes, which led to “adipocyte dedifferentiation” and ultimately to an accumulation of fibroblast-like preadipocytes and cancer progression. Another in vitro study showed that the exomes secreted by preadipocytes (3T3L1 cells) influenced the differentiation, stemness, and migration of the cancer cells through miR-140/SOX2/SOX9 axis [53], which promote the progression of cancer.

A high density of tumor-associated macrophages (TAMs) which resembled M2 macrophages in cancer often correlated with poor prognosis [54]. TAMs were reported to be associated with poor prognostics for colorectal cancer [55]. Consistently, our results showed macrophage M2 were lower for radiosensitive than radioresistant patients.

T cell CD4 memory resting was reported to correlate with poor outcome in colorectal cancer [56]. We found that T cell CD4 memory resting was favorable in predicting RT outcome. T cell CD4 memory resting can generate secondary effector CD4 T cells, with a much higher secondary response to neoantigen than primary effector CD4 T cells, which may be relevant to its favorable role in RT efficacy. T cell CD4 memory resting has a positive association with neoantigen load in many cancer types [57]. The discrepancy of our findings with the previous reports in outcome prognosis may be caused by the different cancer types and treatment modalities adopted.

### Association study between cell type proportions and cytolytic activity and between themselves

Among the eight cytolyic and immune genes tested in this study,i.e., GZMA, GZMB, PRF1, IFNG, CXCL10, SCGB2A2, PDCD1, SCGB2A1, our results demonstrated that only GZMA was significantly different, specifically lower in responsive group (R vs. NR, 43.3 ± 21.1 vs. 90.9 ± 89.6, *p* = 0.006, t-test with unequal variance). Consistently, recent research showed that GZMA promote the colorectal cancer progression by inducing IL-6 production through NF-κB and activating pSTAT3 in colorectal cancer [58]. Similarly, results showed that the cytolytic activity (CYT) represented by the geometric mean of the expression of GZMA and PRF1 gene was significantly higher in nonresponders (NR) than in responders (R) (143.4 ± 77.5 vs. 99.4 ± 42.4, *p* = 0.022). Contrary to our results, other study showed higher CYT is a favorable prognosis for colorectal cancer [38]. Since our result showed no significant difference of PRF1 expression in R versus NR group, the difference in CYT is due to GZMA, which may be an unfavorable factor as reported in [58].

To characterize the cytolytic activity and functionality of the six significant cell type proportions, correlation analysis was performed between proportions of the tissue infiltrated cell subsets and the expression of cytolytic activity related genes including GZMA, GZMB, PRF1, IFNG, and CXCL10, where GZMA, GZMB and PRF1 are expected to be produced by cytolytic T cells and NK cells. Our results demonstrate consistency with their biological and favorable roles in RT outcome. CD4^+^ T cell proportions were most significantly and positively associated with interferon-gamma gene expression. This was consistent with the reports of prolonged neoadjuvant chemoradiotherapy leading to higher CD4^+^ T cells and higher IFN gamma level [59]. As for unfavorable predicting factors, proportions of preadipocyte were most negatively associated with perforin gene expression. Proportions of macrophage M2 negatively correlate with PRF1 and IFNG although not reach a significant level. As unfavorable factors in predicting RT outcome, CD8^+^ T cells only show a marginal significant correlation with the expression of PRF1 and INFG. Consistent with [49] in terms of similar granzyme B expression in higher and lower CD8^+^ T cells distributed normal and cancer tissues reflecting repressed cytolytic activity. We propose that the CD8^+^ T cells are cancer-educated with changed functions in rectal cancer microenvironment. Adipocyte and T cells CD4 memory resting were negatively and significantly associated with IFNG and PRF1 expression, respectively. The relevance of this relationship with the rectal cancer RT outcome needs further study. An anti-intuitive positive correlation between T cell CD4 memory resting and M2, and a negative correlation between proportions of adipocytes and CD4^+^ T cells were also found. The functional interpretation of these correlations need further study.

### Stratification of T cell subtypes

We further stratified the T cells into cytotoxic, exhausted, and inflammatory subtypes. The representative T cell cytotoxic include activated CD8^+^ T cells and nature killer T cells. The inflammatory subtypes include CD8^+^ T effector memory cells. We collected cell markers from [41] for cytotoxic and inflammatory T cell subtypes and from [42] for exhausted CD8+ T cells, respectively, and applied single sample Gene Set Enrichment Analysis [43]. We then characterized these functional categories on GSE3493. The results from the differential analysis on the cytotoxic, exhausted, and inflammatory subtypes, showed no statistical significant cell subtypes except activated CD8^+^ T cells (*p* = 0.038), lower in RT responsive groups, which was consistent with the results from TIMER, where the total CD8^+^ T cells was lower in RT responsive groups. The levels of the exhausted CD8^+^ T cells and these T cells inflammatory subtypes may not be relevant to the response to RT for rectal cancer, whereas the activated and total level of CD8^+^ T cells may be relevant. The mechanism behind it needs further study.

### Immune cell type based RT response prediction

We developed an SVM model based on significant immune cell type proportions only. Results from the leave one out cross-validation demonstrated that this model was better in performance compared with the SVM models based on previously reported RT outcome signatures of 33 probes. The AUC of 0.77 obtained from the leave one out cross-validation on the RT dataset has a 95% confidence interval with lower and upper bound values of 0.63 and 0.86 respectively, suggesting it was significantly better than random guess with *p* < 0.05. This AUC score is not satisfactory for clinical application. However, it is significantly better than random guess with a 95% confidential intervals of [0.632, 0.857], which is meaningful for a machine learning method.

High dimensional data with a small number of samples, which is common for clinical gene expression data, can lead to over-fitting and biased machine learning (ML) performance estimates [45]. However, both the SVM nested cross-validation (CV) and the SVM parameter tuning partial nested CV produced robust and unbiased performance estimates regardless of the small sample size. Our study also demonstrated that SVM parameter tuning partially nested leave one out cross-validation (LOOCV) where the *t*-test feature selection was conducted on pooled training and test data resulted in similar performance with the SVM nested LOOCV where the *t*-test feature was conducted on training data only. That suggested that our LOOCV was not over-fitting.

### Association between proportions of peripheral blood immune cell subunits and RT prognosis

Finally, we compared the peripheral blood leukocyte subsets between progressive diseases and those with stable disease on the clinical data obtained from our institution (Hefei Cancer Hospital, Chinese Academy of Sciences) from 2018 through 2019. Higher CD4^+^/CD8^+^ ratios of peripheral blood were still favorable for the prognosis of radiotherapy and regular treatment for rectal cancer patients based on our combined institutional peripheral blood data from 2018 through the March of 2021. Therefore, there are consistency between compositions of peripheral blood immune subsets and tissue infiltrated immune cells. The results may suggest that the compositions of immune cell subsets in peripheral blood and cancer immune microenvironment are predictive factors for RT outcome for rectal cancer.

## Conclusion

Although larger datasets are required for further validation, our results indicate that tissue residue/infiltrated immune/stromal cell types in pre-radiotherapy bulk cancer tissue are potential predictive markers for response to preoperative RT for rectal cancer. We show their consistency with the peripheral immune cell types in the prognosis of the RT response for rectal cancer in our independent dataset. This study suggests the possible important functions of pre-infiltrated/resident immune/stromal cells in efficacy of RT and as potential combinational immunotherapy drug targets, and peripheral blood cell composition as potential low-invasive predictive markers.

## Supplementary Information

**Additional file 1.** Statistically significant cell types from cell type deconvolution based on GSE3493.

**Additional file 2.** Statistically significant cell types validation with pooled dataset of GSE3493 and GSE35452.

**Additional file 3.** SVM model evaluation, the clinical statistics of the peripheral blood FACS data of RT patients, and R versus NR comparison of the FACS data.

## Data Availability

The dataset supporting the conclusions of this article is included within the article and its additional file. GSE3493 is available at https://www.ncbi.nlm.nih.gov/geo/query/acc.cgi?acc=GSE3493; GSE35452 is available at https://www.ncbi.nlm.nih.gov/geo/query/acc.cgi?acc=GSE35452.

## Abbreviations

ACK: Buffered ammonium chloride
BMDCs: Bone marrow derived cells
CAF: Cancer associated fibroblast
CIBERSORT: Cell-type identification by estimating relative subsets of RNA transcripts
CV: Cross validation
CXCL10: Chemokine 10
CYT: Cytolytic activity
DC: Dendritic cells
ECM: Extra cellular matrix
FACS: Fluorescence-activated cell sorting
FN: False negatives
FPR: False positive rate
GEO: Gene expression omnibus
GZMA: Granzyme A
GZMB: Granzyme B
HIF-1α: Hypoxia induced factor-1α
HIS: Hospital information system
ICD: Immunogenic cell death
IFNG: Interferon gamma
IHC: Immunohistochemistry
LOOCV: Leave one sample out cross-validation
ML: Machine learning
NK cells: Nature killer cells
NR: Non-responders
PERF1: Perforin
R: Responders
RECIST: Response evaluation criteria in solid tumors
ROC AUC: Receiver operating characteristic area under the curve
RT: Radiotherapy
SVM: Support vector machine
TGF beta: Tumor growth factor beta
TILs: Tumor infiltrating or resident leukocytes
TIMER: Tumor immune estimation resource
TLS: Tertiary lymphoid structure
TME: Tumor microenvironment
TNF: Tumor necrosis factor
TP: True positives
TPR: True positive rate
xCell: A gene signature-based method for 64 immune and stromal cell type inference
