# Supplementary material for: Association of the tissue infiltrated and peripheral blood immune cell subsets with response to radiotherapy for rectal cancer
Source: BMC Med Genomics. 2022 May 9;15(Suppl 2):107. doi: 10.1186/s12920-022-01252-6 (PMC9082952; doi:10.1186/s12920-022-01252-6)
Supplement: Supplementary file 3 — Additional file 3. SVM model evaluation, the clinical statistics of the peripheral blood FACS data of RT patients, and R versus NR comparison of the FACS data. [file 12920_2022_1252_MOESM3_ESM.docx]

# Features and SVM model evaluation

CIBERSORT has a resolution of 1% for samples with tumor content greater than 50% and 0.1% for samples with tumor content less than 50%. Although two of the eight cell types, macrophage M1 and eosinophils deconvoluted by CIBERSORT are zero inflated, we found that the non-zero-samples of > 1% are not equally distributed. Non-zero-samples for macrophage M1 are all from 35 RT non-responders with an averaged value of 2.55%, while non-zero-samples for eosinophils greater than 1% are all from 11 RT responders. This provided information for SVM prediction, or at least was not harmful for the prediction (data not shown).

**Table S1.** Adopted features for the SVM from differentially distributed proportions of tumor immune/stromal cell types between the non-responsive and responsive rectal cancer tissues.

| **Cell Types** | **Methods** |
| --- | --- |
| CD4^+^ cells | TIMER |
| CD8^+^ cells | TIMER |
| CD4^+^/CD8^+^ ratio | TIMER |
| Preadipocytes | xCell |
| Adipocytes | xCell |
| T cells CD4 memory resting | CIBERSORT |
| Eosinophils | CIBERSORT |
| Macrophages M2 | CIBERSORT |
| Macrophages M1 | CIBERSORT |

SD: standard deviation; (R): propotion of respornsive; (NR): proportion of non-respondive;

We also compared the performance of the eight significant cell type proportions as markers with previously reported 33-probe signature [1] on predicting the response to the RT for rectal cancer based on the same SVM model. Results in Table 1 show that the predictive performance of the eight-cell-type composition markers performed better than the signature genes (see Table S2) in accuracy, area under receiver operating characteristic (ROC) curve (AUC), false positive rate (FPR), specificity (SE), precision (PE) and F-score. The 33-probe signature only has better true positive rate (TPR) of 0.818 than the 8-cell-type composition, 0.727, but the former has a much worse false positive rate (FPR) of 0.429 than the later of 0.200.

Both the SVM partial nested cross-validation (CV) and the SVM nested CV in [2] produced robust and unbiased performance estimates regardless of the small sample size. The SVM partial nested CV means the *t*-test feature selected on pooled training and test data while the SVM parameter tuning performed on training data only. SVM nested CV represents both the *t*-test feature selection and SVM parameter tuning was performed on training data only. We demonstrated that SVM partial nested leave one out CV resulted in similar performance with the nested CV (see Table S2), which suggested no over-fitting for evaluating the performance of predictors and classifiers in this study.

**Table S2** Comparison of the predictive performance of the gene markers and the cell type proportions based on Support Vector Machine Classifiers with balanced data of RT

| **Performance** | **33 probe**  **signature** | **8 cell type compositions** | **8 cell type compositions (with zscore normalizatin)** |
| --- | --- | --- | --- |
| Accuracy | 0.709 | 0.722 | 0.759 |
| AUC | 0.675 | 0.761 | 0.771 |
| TPR | 0.818 | 0.727 | 0.727 |
| FPR | 0.429 | 0.286 | 0.200 |
| Specificity | 0.571 | 0.714 | 0.800 |
| Precision | 0.706 | 0.762 | 0.821 |
| F-score | 0.758 | 0.744 | 0.771 |

**Table S3** Comparison of the predictive performance of the cell type proportions of partially (8-c and 8-c-z) and completely (Ne, Nu, Neu, Neu-z) nested leave one out cross validation based on Support Vector Machine Classifiers with balanced data of radiotherapy.

| **Performance** | **8-c** | **8-c-z** | **Ne** | **Nu** | **Neu** | **Neu-z** |
| --- | --- | --- | --- | --- | --- | --- |
| accuracy | 0.722 | 0.759 | 0.684 | 0.722 | 0.709 | 0.658 |
| AUC | 0.761 | 0.771 | 0.706 | 0.704 | 0.686 | 0.722 |
| TPR | 0.727 | 0.727 | 0.727 | 0.727 | 0.727 | 0.545 |
| FPR | 0.286 | 0.200 | 0.371 | 0.286 | 0.314 | 0.200 |
| Specificity | 0.714 | 0.800 | 0.629 | 0.714 | 0.686 | 0.800 |
| Precision | 0.762 | 0.821 | 0.711 | 0.762 | 0.744 | 0.774 |
| F-score | 0.744 | 0.771 | 0.719 | 0.744 | 0.736 | 0.640 |

8-c, SVM partial nested CV with 8 cell type proportions selected on pooled training and test samples; 8-c-z, as 8-c, but after zscore normalization; Ne, SVM nested leave one out cross-validation (LOOCV) with significant cell type proportions selected on training samples only with *t*-test of equal variance parameter; Nu, as Ne but with *t*-test of unequal variance parameter; Neu, as Ne and Nu but with combined features selected based on *t*-test of both equal and unequal parameters; Neu-z, as Neu, but with zscore normalization.

# The clinical statistics of the peripheral blood FACS data, and R vs NR comparison of the FACS data of RT or chemotherapy patients

**Table S4** Clinical statistics of peripheral blood data of patients with RT outcome of progressive disease vs those with stable disease collected from 2018 through 2019

| **Rectal cancer patients** |  | **Progressive** | **Stable** | **Chi-Square *p* value** |
| --- | --- | --- | --- | --- |
| Age | >=60 | 5 | 1 | 0.04833 |
|  | <60 | 2 | 5 |  |
| Gender | male | 5 | 5 | 0.6115 |
|  | female | 2 | 1 |  |
| Pathologic stage | I-II | 0 | 1 | 0.2165 |
|  | III-IV | 7 | 4 |  |
|  |  |  |  | Binomial test p value |
| Pathologic metastasis | M0 | 0 | 5 | 0.0625 |
|  | M1 | 0 | 0 |  |
| Pathologic nodes | N0 | 0 | 2 | 1 |
|  | N1-N2 | 0 | 3 |  |
| Pathologic tumor size | T1-T2 | 0 | 1 | 0.375 |
|  | T3-T4 | 0 | 4 |  |

Since there is a significant difference on ages between progressive disease group and stable disease group (p<0.05), we further performed the differentially analysis between age groups >= 60 vs. <60. No significant difference were found, indicating the differences were not due to the age difference.

**Table S5** Comparison of the immune cell subsets in peripheral blood of patients older than 60 years with that of patients younger than 60 years collected from 2018 through 2019.

| **Cell types** | ***t*-test (*p*-value)** | **Age >= 60** | **Age < 60** |
| --- | --- | --- | --- |
| CD3-CD19+ | 0.362 | 4.81±1.36 | 8.26±4.16 |
| CD3+CD4+ | 0.33 | 24.5±15.2 | 36.9±18 |
| CD3+CD8+ | 0.747 | 26.9±7.14 | 27.3±8.77 |
| CD3-CD16+CD56+ | 0.428 | 27.1±8.29 | 19.6±13.5 |
| CD3+ | 0.632 | 63.3±4.84 | 69±15.1 |
| CD4/CD8 | 0.189 | 0.914±0.622 | 1.54±0.923 |

**Table S6** Clinical statistics for patients of chemotherapy with progressive disease vs those with stable disease collected from 2018 through 2019

| **Chemotherapy** | | **Progressive** | **Stable** | **Chi-Square p value** |
| --- | --- | --- | --- | --- |
| Age | >=60 | 7 | 18 | 0.4519 |
|  | <60 | 2 | 10 |  |
| Gender | female | 4 | 12 | 0.9334 |
|  | male | 5 | 16 |  |
| PS | I-II | 0 | 1 | 0.5251 |
|  | III-IV | 9 | 22 |  |
| PM | M0 | 0 | 5 | 0.2357 |
|  | M1-M2 | 1 | 3 |  |
| PN | N0 | 0 | 1 | 0.7077 |
|  | N1-N2 | 1 | 7 |  |
| PT | T1-T2 | 0 | 0 | - |
|  | T3-T4 | 1 | 8 |  |

**Table S7** Comparison of the proportions of peripheral immune cell subsets between patients of progressive disease and stable disease of the rectal cancer patients of chemotherapy collected from 2018 through 2019

| **Chemotherapy** | ***P* value** | **Progression (mean±sd)** | **Stability (mean±sd)** |
| --- | --- | --- | --- |
| CD3-CD19+ | 0.467 | 4.56±3.11 | 3.61±3.42 |
| CD3+CD4+ | 0.649 | 25.8±13 | 23.7±11.4 |
| CD3+CD8+ | 0.0011 | 39.3±10.3 | 27.2±8.37 |
| CD3-CD16+CD56+ | 0.0038 | 18.8±6.51 | 32.6±12.7 |
| CD3+ | 0.0165 | 71.8±7.28 | 59.1±14.4 |
| CD4/CD8 | 0.322 | 0.736±0.542 | 0.908±0.416 |

**Table S8** Age and sex statistics for all 255 rectal cancer hospitalized patients with progressive disease vs those with stable disease collected from 2018 through March, 2021

| **Rectal cancer patients** | **Progressive** | **Stable** | **Chi-Square p value** |
| --- | --- | --- | --- |
| >=60 | 46 | 99 | 0.1124 |
| <60 | 25 | 85 |  |
| female | 28 | 80 | 0.5582 |
| male | 43 | 104 |  |

**Table S9** Comparison of the proportions of peripheral immune cell subsets between patients of progressive disease and stable disease of all rectal cancer patients from 2018 through March of 2021.

| **Cell Types** | ***t*-test**  **(equal variance)** | ***t*-test**  **(unequal variance)** | **Wilcox test** | **Stable** | **Progressive** |
| --- | --- | --- | --- | --- | --- |
| CD3^+^ CD4^+^ | 0.0204 | 0.0199 | 0.0172 | 31±13 | 26.8±12.7 |
| CD3^+^CD8^+^ | 0.000113 | 0.000277 | 0.000527 | 26.4±9.87 | 31.9±10.9 |
| CD4^+^/CD8^+^ | 0.00242 | 0.000649 | 0.000281 | 1.4±1.01 | 0.998±0.759 |

**References**

1. Watanabe T, Komuro Y, Kiyomatsu T, Kanazawa T, Kazama Y, Tanaka J, Tanaka T, Yamamoto Y, Shirane M, Muto T *et al*: **Prediction of sensitivity of rectal cancer cells in response to preoperative radiotherapy by DNA microarray analysis of gene expression profiles**. *Cancer research* 2006, **66**(7):3370-3374.

2. Vabalas A, Gowen E, Poliakoff E, Casson AJ: **Machine learning algorithm validation with a limited sample size**. *PLoS One* 2019, **14**(11):e0224365-e0224365.
